# Correction to: Cross-national comparison of psychosocial well-being and diabetes outcomes in adults with type 1 diabetes during the COVID-19 pandemic in US, Brazil, and Iran

**DOI:** 10.1186/s13098-021-00696-7

**Published:** 2021-07-15

**Authors:** Samereh Abdoli, Monica S. V. M. Silveira, Mehri Doosti-Irani, Paulo Fanti, Katherine Miller-Bains, Elizabeth João Pavin, Edimariz Buin Cardoso, Leila Rafee Vardanjani, Kobra Noorian, Danielle Hessler

**Affiliations:** 1 College of Nursing, University of Tennessee, 1200 Volunteer Blvd Rm 155, Knoxville, TN 37996 USA; 2 Faculty of Medical Sciences, University of Campi-nas, Rua Tessália Vieira de Camargo, 126. Cidade Universitária Zeferino Vaz, Campinas, São Paulo 13083-887 Brazil; 3 School of Nursing and Midwifery, Shahrekourd University of Medical Sciences, Shahrekord, Iran; 4 Assessment and Evaluation, Oak Ridge Associated Universities, Oak Ridge, TN 37831 USA; 5 Endocrinology Division, Department of Internal Medicine, Faculty of Medical Sciences, University of Campinas, Rua Tessália Vieira de Camargo, 126. Cidade Universitária Zeferino Vaz, Campinas, São Paulo 13083-887 Brazil; 6 Clinical Psychologist, Faculty of Medical Sciences, University of Campinas, Rua Tessália Vieira de Camargo, 126. Cidade Universitária Zeferino Vaz, Campinas, São Paulo 13083-887 Brazil; 7 Department of Family and Community Medicine, University of California, 500 Parnassus Avenue, San Francisco, CA 94117 USA

## Correction to: Diabetol Metab Syndr (2021) 13: 63 https://doi.org/10.1186/s13098-021-00681-0

In this article [1] the author name Kobra Noorian was incorrectly written as Kobra Noorjan. Furthermore, the affiliation details for Leila Rafiee Vardanjani were incorrectly given as Department of Family and Community Medicine, University of California, 500 Parnassus Avenue, San Francisco, CA 94117, USA but should have been School of Nursing and Midwifery, Shahrekourd University of Medical Sciences, Shahrekord, Iran. In Fig. 1 of this article, in the last box, the number of participants for Brazil should have appeared as shown below.

**Fig. 1:**
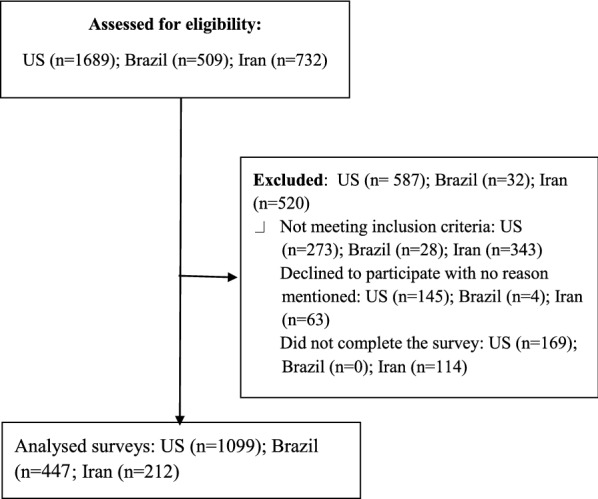
Process of study recruitment

The original article has been updated.
